# Dynamic Bayesian Belief Network for long-term monitoring and system barrier failure analysis: Decommissioned wells

**DOI:** 10.1016/j.mex.2021.101600

**Published:** 2021-12-09

**Authors:** Mei Ling Fam, Xuhong He, Dimitrios Konovessis, Lin Seng Ong

**Affiliations:** a Engineering Cluster, Singapore Institute of Technology, Singapore; b Vysus Sweden AB, Sweden; c Mechanical and Aerospace Engineering, Nanyang Technological University, Singapore; d University of Strathclyde

**Keywords:** Offshore decommissioning, Long-term monitoring, Dependent failures, Bayesian Belief Networks, Common-cause failures

## Abstract

There is increasing interest to consider dependent failures and human errors in the offshore industry. Permanently abandoned wells dot most of the subsea environment. The nature of a well plugging and abandonment (Well P&A) run - usually the lowest-cost contractor engaged to plug several wells tapping the same reservoir makes it an ideal case study for incorporating failures based on common causes. The heavy use of operators during a cementing job also provides the case for analysis of human error in such tasks. One proposed method to analyse the above-mentioned is the use of Bayesian Belief Networks to achieve the following objectives (1) to capture better estimates of a well PA event by incorporating dependencies, and meet regulatory requirements by authorities; and (2) to use the same model to provide long term monitoring of a group of wells linked by common dependencies. This model has not only captured the dependencies of multiple variables, but also projected it in a dynamic manner to provide a risk profile for the next decade where well integrity failure is likely to happen.

• Proposed adapted method capture better estimates of a well PA event by incorporating dependencies

• Method allows for extension of model to long term monitoring of a group of wells linked by common dependencies

## Graphical abstract

**Figure:**
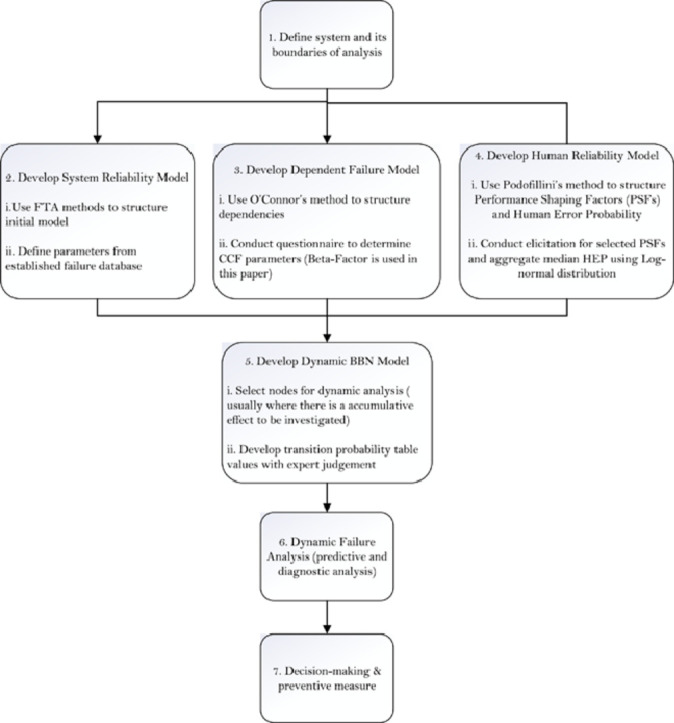

Specifications Table

**Table:** 

**Subject Area**	Energy
More specific subject area:	*Offshore decommissioning, well plugging and abandonment*
Method name:	*Dynamic Bayesian Belief Network for long-term monitoring and system barrier failure analysis*
Name and reference of original method:	*Original methods:* *1. A. O'Connor, A. Mosleh, A general cause based methodology for analysis of common cause and dependent failures in system risk and reliability assessments, Reliability Engineering and System Safety 145 (2016) 341– 350, ISSN 09518320, doi: 10.1016/j.ress.2015.06.007, URL ttp://dx.doi.org/10.1016/j.ress.2015.06.007.* *2. Y. Chang, X. Wu, C. Zhang, G. Chen, X. Liu, J. Li, B. Cai, L. Xu, Dynamic Bayesian networks based approach for risk analysis of subsea wellhead fatigue failure during service life, Reliability Engineering and System Safety 188 (June 2018) (2019) 454–462, ISSN 09518320, doi: 10.1016/j.ress.2019.03.040* *3. L. Podofllini, L. Mkrtchyan, V. N. Dang, Aggregating expert-elicited error probabilities to build HRA models, in: Safety and Reliability: Methodology and Applications, Taylor & Francis Group, London, 2015, pp. 1083-1091.* *4. S. Hauge, Å. S. Hoem, P. Hokstad, S. Håbrekke, M. A. Lundteigen, ommon Cause Failures in Safety Instrumented Systems: Beta-factors and equipment specific checklists based on operational experience, Tech. Rep., Trondheim, doi: 978-82-14-05953-3, 2015.* 5. *A. O. Babaleye, R. E. Kurt, F. Khan, Safety analysis of plugging and abandonment of oil and gas wells in uncertain conditions with limited data, Reliability Engineering and System Safety 188 (August 2018) (2019) 133– 141, ISSN 09518320, doi: doi:10.1016/j. ress.2019.03.027.*
Resource availability:	*Data:* • *Reliability information for the system to be modelled* *Software:* • AgenaRisk: https://www.agenarisk.com/agenarisk-academic • GeNIe: https://download.bayesfusion.com/files.html?category=Academia#

## Method details

The proposed method, though illustrated with a well plugging and abandonment case study is also suitable for any system-based study that includes (i) impact from common causes, (ii) incorporation of Human Reliability Analysis (HRA) and (iii) long-term monitoring. Common Cause failure models and HRA models are specialized risk models, where the types of model proposed in this paper has been best analysed for application in a Dynamic Bayesian Belief Network. A Dynamic Bayesian Belief Network is useful for modelling elements of interest with time, and for allowing dependencies to be linked across all time slices. The first feature allows accumulative fatigue or stress in cement to be considered due to changing wellbore pressure over the years. The second feature allows common dependencies to be modelled, such as the same human operator conducting improper centralization during cementing operations for all wells, affecting the fit of the cement with respect to the well bore. Notably, well leaks do not appear in insolation, thus the same dependencies can extend from Well 1 to Well 2 in the modelling process. Some limitations are that the probability of an event is independent of its history, but only depends upon its immediately previous state. This implies the assumptions made at *t*=0 must be sufficiently robust or comprehensive so that it carries on the information to *t*=*i*-1 if we are interested at the state of events at *t*=*i*.

### Bayesian Belief Networks and dynamic Bayesian Belief Networks

**Fig. 1:**
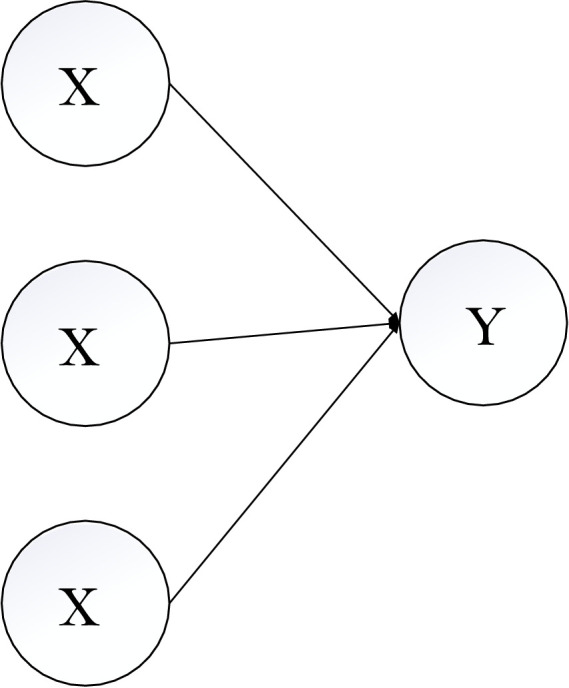
A simplified BBN where X refers to parent nodes and Y a child node.

Bayesian Belief Networks (BBN) are graphical structures (directed acyclic graphs) for representing probabilistic relationships among a number of variables and doing probabilistic inferences with these variables [1]. A BBN consists of nodes, arcs and probabilities tables to represent a set of random variables. The arcs also define the conditional relationships between the nodes, and the information embedded is represented in a conditional probability table (CPT). Consider a BBN with the parents depicted by X, and a child node depicted by Y in Fig. 1. The node Y can take on different states of *y_i_*, where *i* can refer to the number of states. *y_i_* can take on values; or binary or descriptive states like True, False, Good, Moderate, Poor etc. The joint probability distribution of the network is described by Eq. (1):

(1) $$P\left(y_{i}\right)=\Pi_{i=1}^{3}P\left(y_{i}|{x_{\mu }}_{\left(i\right)}\right)$$

where *µ*(*i*) refers to the parents of the node of interest *y_i_*.

The conditional relationship of *y_i_* is represented by its parents: *P* (*x*_1_*, x*_2_*, x*_3_).

Eq. (1) can be expanded by chain rule to:

(2) $$P\left(y_{i}\right)=P\left(y_{i}|x_{0},x_{1},x_{2}\right)\cdot P\left(x_{0}\right)P\left(x_{1}\right)P\left(x_{2}\right)$$

Uncertainties can be presented in a BBN, such as uncertainties of the annular fit of the cement plug in varying levels. The term *y_i_* can take on several states such as ’severely’, ’moderately’ or ’unlikely compromised’. In the case of human reliability analysis, a continuous distribution is considered, and other statistical parameters can be evaluated such as error factors or confidence intervals.

**Fig. 2:**
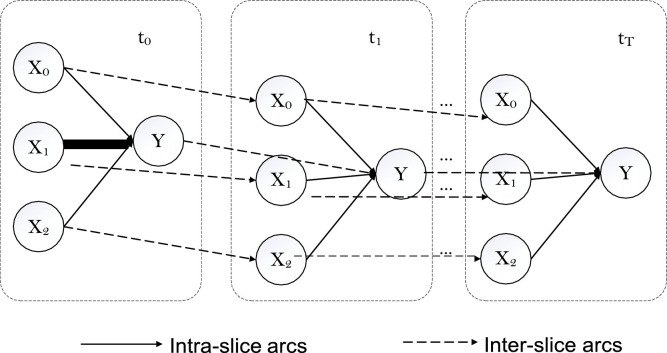
A dynamic BBN over T time slices.

In order to consider a BBN temporal evolution, a dynamic BBN can be employed to explicit model changes over time. This can be represented by integrating the traditional BBN with a discrete-time Markov model. Fig. 2 illustrates how time slices are considered to model the system's discrete temporal changes. The existing time step is represented by *t*_0_, and the subsequent steps *t*_1_, *t*_2_, *t_T_* . The relationships between variables at successive time steps are represented by the inter-slice arcs, $Y^{t0}\to Y^{t1}$. Thus the conditional relationship of *y^t^*1 is increased to *P* (*x^t^*^1^*, x^t^*^1^*, x^t^*^1^*, y^t^*0). The resulting joint probability of the DBN over 2 time slices is defined by (3):

(3) $$P\underset{}{\left({y_{i}}^{t=1}\right)}=P\underset{}{\left(y_{i}{}^{t=1}|x_{0},x_{1},x_{2},{y_{i}}^{t=0}\right)}\cdot P\left(x_{0}\right)P\underset{}{\left(x_{1}\right)}P\left(x_{2}\right)P\left({y_{i}}^{t=0}\right)$$

### Data used in modelling

There are two types of data used to populate the probability tables of the BBN. The first type is considered objective information, usually collated statistically and obtained from databases such as OREDA [2] or from the industry. Such information is usually ideal for describing reliability values of equipment components. Such databases usually do not have information on scarce equipment, or equipment which are hardly studied on due to the lack of access, or that it is lowly prioritized due to economical factors. Decommissioned equipment are usually not looked at again after the primary monitoring period. This scarcity in information thus warrant the need for subjective information.

Subjective information, also known as expert judgement or elicitation is used to fill such data gaps. There are numerous methods of aggregating expert opinions and is widely used in quantitative risk analysis in consequence modelling in offshore risk analysis. Expert judgement is also extensively used in human reliability analysis in nuclear probabilistic risk assessment.

**Fig. 3:**
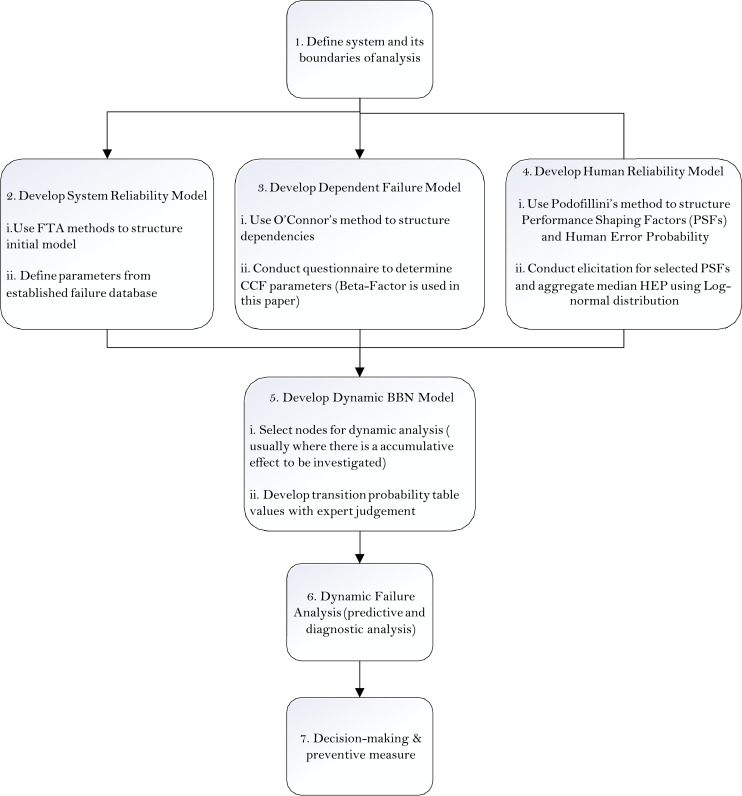
Framework of the proposed methodology.

**Fig. 4:**
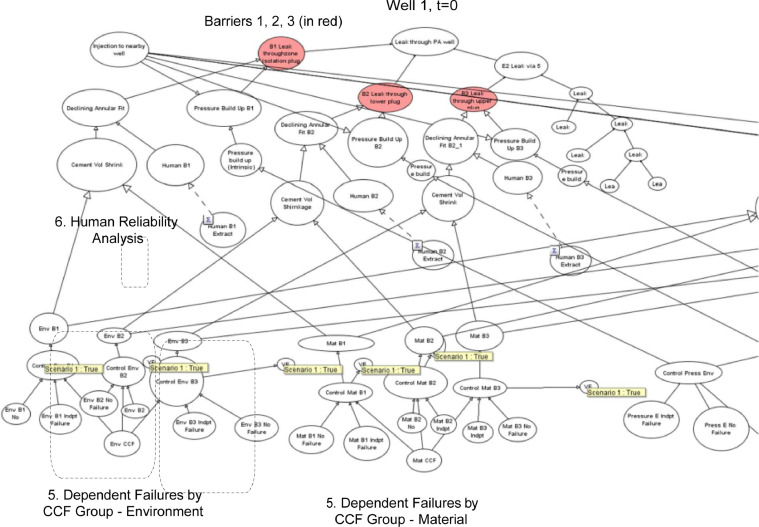
Overall BBN model for PA Well 1, t=0. PA Well 2 is linked also by the CCF groups and HRA and are not shown in this picture due to the high number of nodes. See Fig. (6) for how PA Well 2 is linked by the dependencies.

The proposed methodology for the dynamic risk assessment of an event of interest, for e.g. leaks through a PA well is shown in Fig. 3 and involves the following components:

1. Defining the system
  The first step is on defining the context of the risk assessment. This step usually entails defining the boundary of analysis, for e.g. the number of wells of interest and what types of well. Operational information is usually also required, such as how the plugging and abandonment of wells are carried out, and which guidelines, codes and other legal requirement are applicable to the context. At this stage, workshops are usually carried out to provide an initial identification of hazards of interest.
2. Development of the system reliability model
  The skeleton of the model can be initiated with system reliability analysis to identify links of interest, in this case, the leak paths from where an overall leak through a PA well is realised. Relevant parameters need to be identified as well, such as the failure probabilities of the basic events (termed as nodes in a BBN). The relationships between the parent and child nodes are mostly defined as OR and AND gates, thus the Conditional Probability Tables (CPT) are defined with respective ’1’s and ’0’s to mimic the respective gates [3]. Other kinds of logic gates are used in the model as well, such as the NOISYOR gates from the nodes leading to the ’Top Event’ - Leak probability of the PA well. The NOISYOR gate can be described as the quantification of the impact of each causal factor on the node of interest (Leak through PA Well) independently of considering all of the combinations of states of the other parents [4]. The main benefit is that it usually represents the fault mapping scenario (for *e.g.* it is valid that any of the failure of barriers: B1, B2 and B3 (see Fig. 4 for nodes in red) can lead to a leak through the PA well, independent of each other) while significantly reducing the CPT elicitation burden. The results (leak probability) obtained is in between that derived from an OR and AND gate. NOISYOR gates have also been used to model static well PA where there is uncertainty of data [5] and in O'Connor and Mosleh [6]’s common cause failure models.

**Fig. 5:**
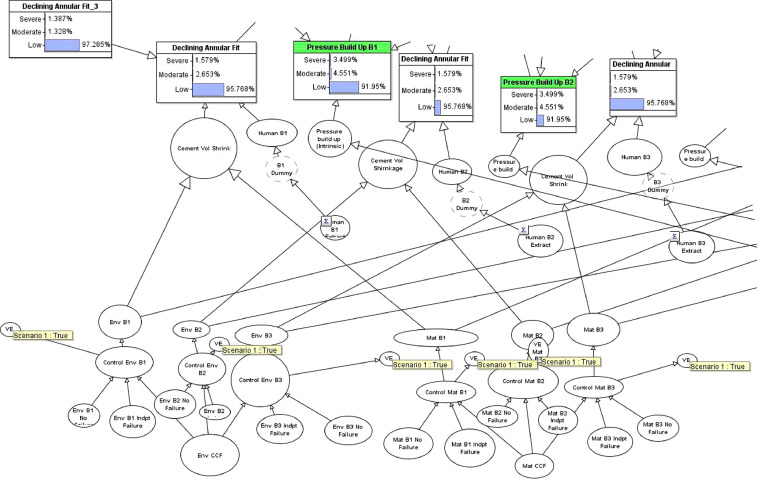
BBN model of common cause failure.
  Same values used in the model are summarised below in Supplementary material and/or information (see Table 1):
3. Development of the dependent failure model
  After the development of the skeletal model, greater level of details can be applied to the nodes identified to have common links. There are numerous commmon cause failure models, and it is proposed that the Beta-Factor model is to be used due to the ease of application, and the recommendation by a Norwegian SINTEF guideline [7] furnished with operational offshore experience ’Common Cause Failure in Safety Instrumented Systems’. The method of incorporating mutually exclusive nodes is documented in Fenton et al. [8]’s work while the application of a common cause failure model in a BBN is highlighted in [6]. The model used by O'Connor [14] is an Alpha-Factor common cause model used in risk assessment of nuclear power plant; however the model in this paper is adapted to a Beta-Factor model with supported use in the offshore industry by Hauge et al. [7]. The common cause failures are often grouped by the context in which the dependencies exists, in this case, the subsea well condition (temperature, pressure, presence of corrosive gases) is one such dependency group. Questionnaire methods can be used to define the Beta-Factor parameters [7,9]. In this paper, it is modelled with one other well (PA Well 2) belonging to the same dependency group as PA Well 1 (See Fig. 6).

**Fig. 6:**
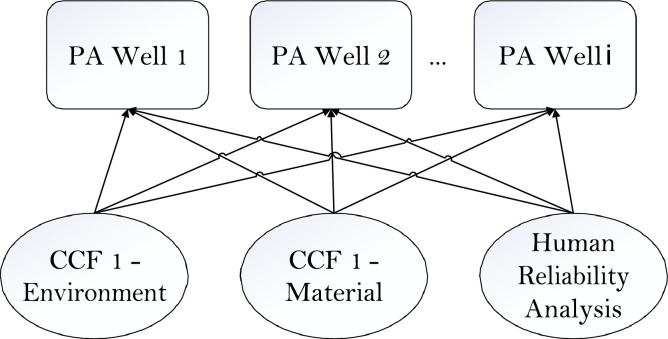
Conceptual model of PA Wells belonging to the same dependency group. PA Well 2,...*i* is linked also by the same CCF groups and HRA as in PA Well 1.
  The sample CCF values are summarised below (see Table 2 and Fam et al. [9]) and are fed into the events of interest.
4. Development of the HRA model
  The HRA part (see Fig. 7) of the model is to link operator dependent actions (such as poor cementing technique) across the wells conducted in a batch operation through established HRA models. The HRA model is also a part of the dependency analysis. There are multiple established HRA models used in the nuclear industry. The chosen model is by Podofillini et al. [10] as it is adapted for use in a BBN. The HRA model developed in this paper stems from the work in [11]. The elicitation of conditional probability tables are found in [11] and in Podofillini et al. [10]. The performance shaping factors [12] are worked through and shortlisted for use in a well PA context.

**Fig. 7:**
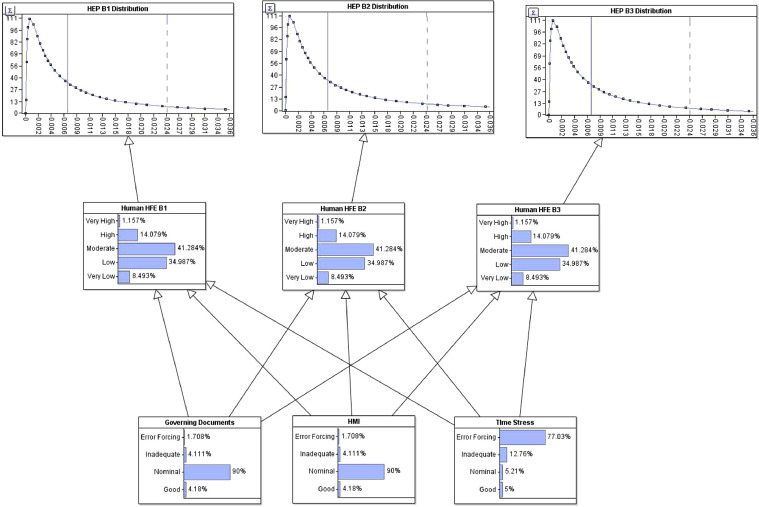
BBN model of human error probability.
5. Development of the Dynamic BBN model
  The development of the Dynamic BBN model is the section of the model where there is interest in investigating the performance of barrier over time, in this case, the annular fit and the casing strength. The transi- tion CPTs represents the dynamic nature of the risk event over time with respect to the discrete-time Markov model. The transition CPTs are de- fined with expert judgement in a manner similar to the method proposed in Table 1 of Chang et al. [13].
  The tables (see Tables 3–5) are elicited from expert judgement and are presented in the section Supplementary material and/or information for reference.
6. Predictive and Diagnostic Analysis
  The nature of a BBN allows for a predictive analysis, such as the prediction of the leak failure probability in a forward-manner, from the nodes up to the top event of interest. Diagnostic analysis can also be conducted by reflecting observations in a particular well, in a particular time slice and the information can be propagated to other nodes (through the defined dependencies), and ultimately update the leak failure.
7. Decision-making and preventive measures
  With information on the most influencing factors, or weakest link, prevention measures can be undertaken to improve the risk profile.

## Declaration of Competing Interest

The authors declare that they have no known competing financial interests or personal relationships that could have appeared to influence the work reported in this paper.

## Appendix Supplementary materials

Image, application 1
